# Antimicrobial Resistance Patterns and Dynamics of Extended-Spectrum β-Lactamase-Producing Uropathogenic *Escherichia coli* in Cusco, Peru

**DOI:** 10.3390/antibiotics10050485

**Published:** 2021-04-22

**Authors:** Steev Loyola, Fátima Concha-Velasco, Jimena Pino-Dueñas, Nancy Vasquez-Luna, Paola Juarez, Carlos Llanos, Guillermo Salvatierra, Jesus Tamariz, Andres G. Lescano

**Affiliations:** 1 Laboratorio de Resistencia Antibiótica e Inmunopatología, Facultad de Medicina, Universidad Peruana Cayetano Heredia, Lima 15102, Peru; paola.juarez.c@upch.pe (P.J.); daniel.llanos.bio@gmail.com (C.L.); jesus.tamariz@upch.pe (J.T.); 2 Departamento de Apoyo al Diagnóstico, Hospital Antonio Lorena, Cusco 08001, Peru; fatima.concha.v@gmail.com (F.C.-V.); jimenaedith@hotmail.com (J.P.-D.); nancyvalu@hotmail.com (N.V.-L.); 3 Laboratorio de Genómica Microbiana, Departamento de Ciencias Celulares y Moleculares, Facultad de Ciencias y Filosofía, Universidad Peruana Cayetano Heredia, Lima 15102, Peru; gsalvatierrar@gmail.com; 4 Emerge, Emerging Diseases and Climate Change Research Unit, School of Public Health and Administration, Universidad Peruana Cayetano Heredia, Lima 15102, Peru

**Keywords:** urinary tract infections, *Escherichia coli*, β-lactamases, β-lactam resistance, extended-spectrum β-lactamase

## Abstract

Urinary tract infections (UTIs) are a common human infection. Antibiotic resistance in extended-spectrum β-lactamase (ESBL)-producing uropathogenic *E. coli* (UPEC) is a major therapeutic challenge due to limited treatment alternatives. The aim was to characterize the antimicrobial resistance (AMR) and dynamics of ESBL-producing UPEC isolates from UTI cases seen at a local hospital in Cusco, Peru. Ninety-nine isolates from respective patients were characterized against 18 different antibiotics. Latent class analysis (LCA) was used to evaluate the dynamics across the study time according to resistance patterns. The median age of patients was 51 years old, and nearly half were women. ESBL-producing UPEC isolates were slightly more frequent in outpatient services than emergency rooms, and there were higher resistance rates in males compared to females. Half of the ESBL producers were resistant to aminoglycosides and nitrofurantoin. Cefoxitin and fosfomycin resistance was 29.3% and 14.1%, respectively. Resistance to carbapenems was not observed. All isolates were multidrug-resistant bacteria, and 16.2% (16/99) were also classified as extensively drug-resistant bacteria. The resistance patterns varied across the study time and differed regarding sex and healthcare service. The study revealed high levels of AMR to commonly used antimicrobials and a dynamic circulation of ESBL-producing UPEC isolates with varying resistance patterns.

## 1. Introduction

Urinary tract infections (UTIs) affect more than 150 million people/year worldwide [1]. In the United States alone, UTIs result in an estimated seven million and one million ambulatory and emergency care visits, respectively, and account for ~USD 3.5 billion/year in costs, including healthcare and time missed from work [2,3]. In Peru, UTIs are not reported in routine epidemiological surveillance, and no accurate incidence estimates are available [4]. In the context of antimicrobial resistance surveillance, this lack of information translates into a lack of evidence that potentially could be used to improve infection management.

*Escherichia coli* strains are the most frequent etiological agent of UTIs worldwide, accounting for 70–95% of these infections [5]. Although some UTIs do not require antibiotic treatment, they are one of the most common indications for antimicrobials, and the unregulated use of antibiotics has led to the emergence of multidrug-resistant UTI-causing bacteria [6]. Thus, there is a need for the prudent use of antimicrobials in order to avoid the emergence of antimicrobial resistance.

Extended-spectrum β-lactamase (ESBL)-producing uropathogenic *E. coli* (UPEC) represent a major health concern due to their resistance to penicillins and cephalosporins [7,8,9]. ESBLs are mainly classified in three classes: TEM, SHV, and CTX-M [10]. The plasmids that encode *bla_ESBLs_* frequently carry other resistance genes with activity against commonly used antibiotics such as aminoglycosides, sulfonamides, and quinolones. Thus, UTIs due to multidrug-resistant ESBL-producing UPEC strains have limited treatment options and represent a major, expanding public health threat [11]. Therefore, the aim of this study was to characterize the antimicrobial resistance and dynamics of ESBL-producing *E. coli* strains isolated from UTIs treated in a public hospital in Cusco-Peru and to explore the association between patients’ and healthcare services’ characteristics to antibiotic resistance rates.

## 2. Results

Ninety-nine ESBL-producing UPEC strains were isolated from the same number of patients between January and December 2017. The isolation rate was similar during all trimesters resulting in 23 to 27 isolates per trimester. The median age of patients was 51 years old (range: 1–92), and nearly half (45%) were women. ESBL-producing UPEC isolates came slightly more from outpatient services than emergency rooms (58.6% vs. 41.4%).

### 2.1. Antimicrobial Resistance Characterization

The isolates showed resistance rates above 80% to ciprofloxacin and trimethoprim/sulfamethoxazole (Table 1). Approximately half of the isolates were resistant to aminoglycosides and nitrofurantoin, and one-third of the isolates to cefoxitin and penicillins combined with β-lactamase inhibitors (Table 1). Fosfomycin resistance was 14.1%. All isolates were classified as multi-drug-resistant (MDR) bacteria and 16.2% (16/99) were also classified as extreme drug-resistant (XDR) bacteria, but we did not observe any pan-drug-resistant (PDR) bacteria as all isolates were susceptible to carbapenems by phenotypic methods.

Among the 58 ESBL producers randomly selected for *bla* gene testing, 42 (72.4%) isolates harbored enzymes of the *bla_CTX_*_-M_ group 1, 15 (25.9%) of the *bla_CTX-M_* group 9, 22 (37.9%) of the *bla_TEM_*. In addition, we detected 18 (31.0%) bacterial isolates that specifically encode the *bla_CTX-M-14_*. Four isolates were negative for all genes tested, and only two isolates were positive for all of them (Table 2).

### 2.2. Factors Associated with Antimicrobial Resistance

Cefoxitin resistance rate was twice as high in ESBL-producing UPEC isolates from females compared to males (42.2% vs. 18.5%, *p* = 0.014, Table 1). No other differences in antimicrobial resistance potentially associated with the patient’s gender, age group, or healthcare service were observed when analyzing individual resistance rates for each antibiotic.

The resistance rate for amikacin increased across the study period (*p* = 0.046, Table 3). Similarly, the resistance rate for cefoxitin increased in the second trimester and remained at high levels since. In contrast, the observed resistance for nitrofurantoin decreased over the study period (*p* = 0.007, Table 3) and was high for fosfomycin only in the first and fourth trimester. No other trends were observed for the evaluated drugs.

### 2.3. Potential Antimicrobial Resistance Phenotypes by Latent Class Analysis (LCA)

Carbapenems, expanded-spectrum cephalosporins, and penicillin categories were excluded from LCA because all ESBL-producing UPEC were carbapenem-susceptible. Similarly, the penicillin + β-lactamase inhibitor category was also excluded because almost all isolates were simultaneously resistant to a cefoxitin. Cefoxitin and the monobactam aztreonam were included in the analysis due to their discriminatory power assessed by LCA.

A three-phenotype (three classes) LCA model fitted the resistance patterns data the best compared to models with more or less phenotypes, although its fit was only slightly better than a four-phenotype model (Table 4); thus, the results from models with three and four phenotypes were initially explored (Table 5). The distribution of the antibiotic resistance rate patterns was slightly similar among the phenotypes identified by the two LCA models. Both models identified a phenotype highly resistant to all antibiotics (3-1 and 4-1, respectively), but the four-phenotype model allowed for better discrimination of resistance patterns for aminoglycosides, fluroquinolones, monobactams, phosponic acids, and nitrofurans compared to the three-phenotype model (Differences > 15%, Table 5). Therefore, four phenotypes with distinct resistant patterns are described in all further analyses.

Based on the 4-phenotype model, we evaluated the relation between patients’ characteristics and phenotypes as well as the dynamics of those phenotypes by trimesters. (Table 6). Phenotype one (4-1) was similarly distributed over the study time (Table 6). In contrast, phenotypes two and four were frequent during the first and second trimesters and then they seemed to be “replaced” by the “emergence” of phenotype three in the third and fourth trimesters (*p* < 0.001). Phenotypes differed regarding the male:female ratios (*p* = 0.007), and the proportion of patients seen in emergency wards was also marginally different across genotypes (*p* < 0.150). Ex post, the largest potential differences by gender were observed in phenotype one versus two (*p* = 0.001). Phenotype 1 was more common in older individuals while genotype 3 was more common in younger cases (*p* = 0.045). Phenotype 4 was more common in emergency visits compared to phenotypes 2 and 3 (*p* = 0.047).

## 3. Discussion

Over one year, all ESBL-producing *E. coli* isolates from patients with suspected UTIs presented a multidrug-resistant phenotype against clinically important antibiotics. Resistance patterns in pathogenic *E. coli* are important for the appropriate selection of therapy for UTIs [12,13]. We found high resistance to trimethoprim-sulfamethoxazole, ciprofloxacin, aminoglycosides, and non-carbapenem β-lactams, in addition to highly dynamic strain circulation with varying resistance patterns. Our findings suggest that antibiotic treatment of UTIs should be preferably based on antimicrobial susceptibility testing to improve clinical outcomes and avoid selection and spread of resistant *E. coli* strains.

While testing is unavailable, fosfomycin seems to be a good candidate for non-complicated UTIs, particularly for MDR/XDR infections [14]. This broad-spectrum antibiotic showed 14.1% to 25% resistance in 2017. Nitrofurantoin has recently gained importance as a first-line antibiotic for non-complicated UTI management [12]. However, only one of the four phenotypes identified in this study was susceptible to nitrofurantoin. Thus, the use of this antimicrobial as a first-line option may need to be reconsidered as a treatment option in the studied hospital. Carbapenems are stable against ESBL-mediated hydrolysis and their broad spectrum makes them the antimicrobial class of choice for complicated UTIs treatment [15]. No resistance to imipenem, ertapenem, and meropenem was found in ESBL-producing UPEC isolates across the study time. However, the use of carbapenems should be appropriate and limited to UTI cases caused by carbapenem-resistant organisms.

The incidence, presentation, and course of UTIs differed between female and male patients due to anatomical differences. In males, because of prostate involvement, it is common to use ciprofloxacin or trimethoprim/sulfamethoxazole to reach therapeutic levels in the prostate [16,17]. Trimethoprim/sulfamethoxazole, fosfomycin, and nitrofurantoin are recommended for female patients. [12] Our results are consistent with previously published observations. For example, MDR UTIs are common among female patients [18], and females and males presented comparable resistance rates for the recommended antibiotics, suggesting that such drug choices were inappropriate for the study population. Low fosfomycin resistance was found among male and female patients, and this drug appears to be a good candidate to treat uncomplicated UTIs. However, fosfomycin resistance varied from 8.7% to 35.7% among the four phenotypes identified by LCA, suggesting that its efficacy may vary importantly across strains.

Cefoxitin resistance was frequent among female patients, consistent with observations from previous reports [19,20,21]. Remarkably, all cefoxitin-resistant isolates clustered into phenotype 4-1, suggesting that cefoxitin could be used for three of the four phenotypes, which represents almost two-thirds of all UTI cases.

Age is another important factor associated with UTI and drug resistance emergence. Older age groups can have high incidence due to genito-urinary atrophy and vaginal prolapse, and UTIs are some of most common infections in elderly populations [22,23]. In this study, we observed that phenotype 4-1 was more frequent among older individuals compared to other phenotypes.

Phenotype 4-1 was similarly distributed across the study period but phenotypes 4-2 and 4-4 circulated mainly in the first and second trimesters and then were apparently replaced by phenotype 4-3 in the third and fourth trimesters. Cusco is considered a highly cosmopolitan city and is known for its cultural background and touristic nature, being a hotspot for millions of tourists annually all over the world. The emergence of a different phenotype (4-3) in the third and fourth trimesters (July–December) took place during/after the northern hemisphere summer and mid-year vacations, the annual peak of international tourism [24]. The increase and variability of antimicrobial resistant organisms is related to population mobility and globalization processes; thus, tourism dynamics in Cusco could be associated with the importation of different drug-resistant microorganisms, facilitating the potential emergence of a different resistance phenotype such as the phenotype 4-3 and broadening the diversity of drug-resistant bacteria [25]. Such hypotheses require further and more in-depth exploration.

Even with a limited sample size, marginally significant differences in phenotype distributions were found between outpatient and emergency patients. Phenotypes 4-2 and 4-3 were more frequent among UTI patients from outpatient services. Phenotype 4-4, which includes isolates resistant to aminoglycosides, trimethoprim /sulfamethoxazole, fosfomycin, and nitrofurantoin, was more frequent in emergency room patients than in outpatients. UTI patients in emergency rooms often presented with comorbidities or recurrent UTIs with previous antibiotic exposures leading to MDR infections [26]. The incomplete molecular characterization of β-lactamase genes limited the detection of additional genetic determinants in a subset of ESBL-producing isolates across the study time. Interestingly, the presence of only the *bla_TEM_* gene in four isolates and the absence of all screened genes in the other four isolates suggests the presence of additional genes that potentially contribute to β-lactam resistance. Additionally, the lack of assessment of virulence markers in UPEC isolates limited our understanding of pathogenicity and proper antimicrobial management. Further studies need to take into account the limitations in order to better understand the epidemiology and phenotype distribution across time.

MDR and XDR *E. coli* are a serious and ever-expanding global threat to the control of UTIs, limiting viable treatment options and demanding the implementation of antibiotic stewardship interventions; clear, strict treatment protocols; and the continued identification of susceptible antimicrobials. Some of the main factors for antimicrobial resistance in low middle-income countries are the unrestricted access to antimicrobials, their misuse when treating non-bacterial infections [27], and inadequate dosage that creates selective pressure and expands MDR bacteria populations [28]. In conclusion, our findings suggest that ESBL-producing UPEC isolates in Cusco, Peru, are highly resistant to most antimicrobials of clinical relevance, although the distribution of resistant strains over time can be highly dynamic with potential seasonal trends. Carbapenems, fosfomycin, and cefoxitin appear to be good candidates to treat UTIs; however, the responsible use of them is critical to control the expansion of this major antimicrobial threat.

## 4. Materials and Methods

### 4.1. Clinical Isolates

The Antonio Lorena hospital is a tertiary public hospital located in Cusco city, in the Peruvian highlands (13°31′30″ S 71°58′20″ W). The hospital’s laboratory routinely receives and analyzes urine samples of patients from the emergency and outpatient services seen due to symptoms clinically compatible with UTIs. Urine samples were obtained by the mid-stream catch collection method, and *E. coli* strains were isolated using MacConkey agar (Thermo Fisher: CM0007) and characterized at the species level using standard microbiological procedures. Briefly, bacteria were identified as *E. coli* by colony morphology, Gram staining, and biochemical reactions based on fermentation.

ESBL production is routinely determined using the Clinical and Laboratory Standards Institute (CLSI) confirmatory test [29] at the hospital’s laboratory in Cusco. All ESBL-producing UPEC isolates were aliquoted and stored using 1.5 mL tubes with 0.5 mL trypticase soy agar (Thermo Fisher: CM0131) at 4 °C and sent quarterly to Universidad Peruana Cayetano Heredia (UPCH) in Lima, Peru, for phenotypic and molecular characterization. The study was approved by UPCH’s ethics committee (SIDISI: 100535), and the hospital also approved the study protocol and its procedures.

### 4.2. Antimicrobial Susceptibility Testing

ESBL production determined by the Antonio Lorena’s laboratory was phenotypically confirmed at UPCH following CLSI standards [29]. All bacterial isolates were tested for antimicrobial susceptibility at UPCH using the Kirby–Bauer disk diffusion method according to the CLSI and included 17 drugs from 11 antimicrobial categories: aminoglycosides (gentamicin and amikacin), penicillins with β-lactamase inhibitors (piperacillin-tazobactam and amoxicillin-clavulanic acid), carbapenems (ertapenem, imipenem and meropenem), expanded-spectrum cephalosporins (ceftriaxone, ceftazidime and cefepime), cephamycin (cefoxitin), fluroquinolone (ciprofloxacin), folate pathway inhibitors (trimethoprim-sulphamethoxazole), monobactam (aztreonam), penicillin (ampicillin), phosphonic acid (fosfomycin), and nitrofuran (nitrofurantoin). The susceptibility results were interpreted according to CLSI cutoffs for each antimicrobial used in this study [29]. The production of carbapenemases was assessed according to previously described phenotypic methods [29,30,31,32]. *E. coli* ATCC 25955 and 35218 were used as quality controls for antimicrobial susceptibility testing.

### 4.3. Molecular Testing for β-Lactamase Genes

A random sample of 58 (58.6%) ESBL-producing UPEC was selected for testing *bla_CTX-M_* group 1 (Forward: 5′-ATG GTT AAA AAA TCA CTG CG-3′; Reverse: 5′-TTA CAA ACC GTC GGT GAC-3), *bla_CTX-M_* group 9 (Forward: 5′-ATG GTG ACA AAG AGA GTG CAA C-3′; Reverse: 5′-TTA CAG CCC TTC GGC GAT G-3′), *bla_CTX-M-14_* (Forward: 5′-AAC ACG GAT TGA CCG TAT TG-3′; Reverse: 5′-TTA CAG CCC TTC GGC GAT-3′), *bla_TEM_* (Forward: 5′-ATG AGT ATT CAA CAT TTC CGT G-3′; Reverse: 5′-TTA CCA ATG CTT AAT CAG TGA G-3′), and *bla_SHV_* (Forward: 5′-ATG CGT TAT ATT CGC CTG TG-3′; Reverse: 5′-GTT AGC GTT GCC AGT GCT CG-3′) genes in single polymerase chain reactions (PCR) as previously described [33,34,35]. The DNA was extracted from one bacterial colony by the thermal shock method in a total volume of 50 µL. Briefly, PCRs were performed in a 50 µL reaction mixture containing 1 µL (250 ng) of DNA extracts using 1 × PCR buffer, 1.0 mM MgCl_2_, 100 mM of each dNTPs, 100.0 pM of each oligonucleotide primer, and 1.0 unit of Taq DNA polymerase (Thermo Fisher: 10342053). The amplification was carried out in a terminal cycler using the PCR conditions described for each target gene [33,34,35]. PCR products were separated by electrophoresis on 1% agarose gel and revealed by RunSafe under the conditions described by the manufacturer [36].

### 4.4. Statistical Analysis

ESBL-producing UPEC with intermediate antibiotic resistance were considered resistant for data analysis purposes. Strains were classified as MDR, XDR, and PDR according to standardized definitions described elsewhere [37]. Briefly, an isolate was classified as MDR if it was non-susceptible to at least one agent in three or more categories and classified as XDR if it was non-susceptible also to at least one agent in all but two or fewer categories. PDR was a classification assigned to isolates with exhibited resistance to all agents tested in this study. LCA was applied to the results of seven antimicrobial categories to identify potential latent resistance phenotypes. The adjusted Bayesian Information Criteria (BIC), entropy, and an enhanced phenotype discrimination were used to evaluate the model fit [38,39], as well as the comparison of resistance levels to each antibiotic between identified groups. LCA model stability was assessed in randomly selected bacterial isolates. Bivariate analyses of factors associated with resistance were performed using Fisher’s exact test. Analyses were conducted using Stata version 15.0 (StataCorp. 2017. Stata Statistical Software: Release 15. College Station, TX, USA: StataCorp LLC.), and significant associations were considered if *p* < 0.05.

## Figures and Tables

**Table 1:** Antibiotic resistance rates of ESBL-producing uropathogenic *E. coli* according to patients’ gender, age groups, and healthcare service.

Category/Agent	Total (*n* = 99)	Gender		*p*-Value	Age Groups (Years)			*p*-Value	Health Care Service		*p*-Value
		Female (*n* = 45)	Male (*n* = 54)		<18 (*n* = 6)	18–60 (*n* = 55)	>60 (*n* = 38)		Emergency (*n* = 41)	Outpatient (*n* = 58)	
	% (*n*)	% (*n*)	% (*n*)		% (*n*)	% (*n*)	% (*n*)		% (*n*)	% (*n*)	
1. Aminoglycosides											
Gentamicin	50.5 (50)	51.2 (23)	50.0 (27)	1.000	50.0 (3)	52.7 (29)	47.4 (18)	0.895	51.2 (21)	50.0 (29)	1.000
Amikacin	46.5 (46)	53.3 (24)	40.7 (22)	0.231	66.7 (4)	50.9 (28)	36.8 (14)	0.249	41.5 (17)	50.0 (29)	0.421
2. Penicillins + β-lactamase inhibitors											
Piperacillin/tazobactam	35.6 (35)	35.6 (16)	35.2 (19)	1.000	50.0 (3)	40.0 (22)	26.3 (10)	0.300	31.7 (13)	37.9 (22)	0.670
Amoxicillin/clavulanic acid	37.4 (37)	31.1 (14)	42.6 (23)	0.298	50.0 (3)	45.5 (25)	23.7 (9)	0.079	43.9 (18)	32.8 (19)	0.296
3. Cephamycin											
Cefoxitin	29.3 (29)	42.2 (19)	18.5 (10)	0.014	50.0 (3)	21.8 (12)	36.8 (14)	0.149	29.3 (12)	29.3 (17)	1.000
4. Fluoroquinolone											
Ciprofloxacin	83.8 (83)	84.4 (38)	83.3 (45)	1.000	83.3 (5)	83.6 (46)	84.2 (32)	1.000	78.1 (32)	87.9 (51)	0.268
5. Folate pathway inhibitor											
Trimethoprim/ Sulfamethoxazole	80.8 (80)	88.9 (40)	74.1 (40)	0.076	83.3 (5)	81.8 (45)	79.0 (30)	0.915	87.8 (36)	75.9 (44)	0.196
6. Phosponic acid											
Fosfomycin	14.1 (14)	11.1 (5)	16.7 (9)	0.565	16.7 (1)	12.7 (7)	15.8 (6)	0.904	17.1 (7)	12.1 (7)	0.563
7. Nitrofuran											
Nitrofurantoin	53.5 (53)	51.1 (23)	55.6 (30)	0.690	66.7 (4)	52.7 (29)	52.6 (20)	0.896	48.8 (20)	56.9 (33)	0.540
XDR *											
Yes	16.2 (16)	24.4 (11)	9.3 (5)	0.055	33.3 (2)	12.7 (7)	18.4 (7)	0.313	14.6 (6)	17.2 (10)	0.788

* Considering eleven categories: aminoglycosides, penicillins + β-lactamase inhibitors, cephamycin, fluoroquinolone, folate pathway inhibitor, monobactam, phosponic acid, nitrofuran, penicillin, carbapenems, and expanded-spectrum cephalosporins. All isolates were MDR, and no isolates were PDR. Resistance to carbapenems was not observed.

**Table 2:** Types of ESBL genes detected among ESBL-producing uropathogenic *E. coli* isolates (*n* = 58).

*bla*_CTX-M_ Group 1	*bla*_CTX-M_ Group 9	*bla*_CTX-M-14_	*bla*_TEM_	*n* (%)
+	−	−	−	18 (31.0)
+	−	−	+	11 (18.9)
+	+	+	−	6 (10.3)
−	+	+	−	6 (10.3)
−	−	−	−	4 (6.9)
+	−	−	+	3 (5.2)
−	−	−	+	3 (5.2)
+	−	+	−	2 (3.4)
+	+	+	+	2 (3.4)
−	−	−	+	1 (1.7)
−	+	+	+	1 (1.7)
−	−	+	+	1 (1.7)
*n* = 42, 72.4%	*n* = 15, 25.9%	*n* = 18, 31.0%	*n* = 22, 37.9%	Total

Note: All the 58 ESBL producers selected were negative for the *bla_SHV_* screening. Positive (+) and negative (−) PCR results were presented for each screened *bla* genes.

**Table 3:** Antimicrobial resistance rates of ESBL-producers across the study time.

Category/Agent	Trimester				*p*-Value
	First (*n* = 25)	Second (*n* = 23)	Third (*n* = 27)	Fourth (*n* = 24)	
	% (*n*)	% (*n*)	% (*n*)	% (*n*)	
1. Aminoglycosides					
Gentamicin	48.0 (12)	39.1 (9)	51.9 (14)	62.5 (15)	0.460
Amikacin	24.0 (6)	47.8 (11)	51.9 (14)	62.5 (15)	0.046
2. Penicillins + β-lactamase inhibitors					
Piperacillin/tazobactam	32.0 (8)	43.5 (10)	25.9 (7)	41.7 (10)	0.521
Amoxicillin/clavulanic acid	26.1 (6)	45.8 (11)	31.0 (9)	47.8 (11)	0.273
3. Cephamycin					
Cefoxitin	8.0 (2)	34.8 (8)	40.7 (11)	33.3 (8)	0.035
4. Fluoroquinolone					
Ciprofloxacin	76.0 (19)	73.9 (17)	92.6 (25)	91.7 (22)	0.146
5. Folate pathway inhibitor					
Trimethoprim/sulfamethoxazole	88.0 (22)	69.6 (16)	81.5 (22)	83.3 (20)	0.451
6. Phosphonic acid					
Fosfomycin	24.0 (6)	0.0 (0)	7.4 (2)	25.0 (6)	0.015
7. Nitrofuran					
Nitrofurantoin	76.0 (19)	65.2 (15)	40.7 (11)	33.3 (8)	0.007
XDR *					
Yes	4.0 (1)	17.4 (4)	25.9 (7)	16.7 (4)	0.171

* Analysis considering eleven categories: aminoglycosides, penicillins + β-lactamase inhibitors, carbapenems, expanded-spectrum cephalosporins, cephamycin, fluoroquinolone, folate pathway inhibitor, monobactam, penicillin, phosponic acid, and nitrofuran. Resistance to carbapenems was not observed. Note: The first trimester was considered from January to March, the second from April to June, the third from July to September, and the fourth from October to December 2017.

**Table 4:** Latent class analysis model fit.

Goodness-of-Fit Criteria *	Number of Classes				
	2	3	4	5	6
Adjusted Bayesian Information Criteria	95.990	82.766	83.650	89.314	95.754
Entropy	0.795	0.696	0.709	0.752	0.764

* Lower values imply better model fit.

**Table 5:** Phenotypes and antibiotic resistance rate patterns of ESBL-producing uropathogenic *E. coli* according to the latent class analysis (*n* = 99).

Phenotypes	AMY	CEP	FLU	FPI	MON	PHA	NIT
	% (*n*)	% (*n*)	% (*n*)	% (*n*)	% (*n*)	% (*n*)	% (*n*)
3-phenotype model							
Phenotype 3-1 (*n* = 38, 38.4%)	100.0 (38)	76.3 (29)	100.0 (38)	89.5 (34)	81.6 (31)	7.9 (3)	73.7 (28)
Phenotype 3-2 (*n* = 31, 31.3%)	93.6 (29)	0.0 (0)	67.7 (21)	100.0 (31)	90.3 (28)	16.1 (5)	0.0 (0)
Phenotype 3-3 (*n* = 30, 30.3%)	33.3 (10)	0.0 (0)	80.0 (24)	50.0 (15)	90.0 (27)	20.0 (6)	83.3 (25)
4- phenotype model							
Phenotype 4-1 (*n* = 39, 39.4%)	100.0 (39)	74.4 (29)	100.0 (39)	89.7 (35)	82.1 (32)	10.3 (4)	74.4 (29)
Phenotype 4-2 (*n* = 23, 23.2%)	17.4 (4)	0.0 (0)	91.3 (21)	47.8 (11)	91.3 (21)	8.7 (2)	87.0 (20)
Phenotype 4-3 (*n* = 23, 23.2%)	100.0 (23)	0.0 (0)	100.0 (23)	91.3 (21)	100.0 (23)	13.0 (3)	0.0 (0)
Phenotype 4-4 (*n* = 14, 14.2%)	78.6 (11)	0.0 (0)	0.0 (0)	92.9 (13)	71.4 (10)	35.7 (5)	28.6 (4)

Note: % (*n*) are presented in rows. AMY: Aminoglycosides, CEP: Cephamycins (Cefoxitin), FLU: Fluoroquinolones (Ciprofloxacin), FPI: Folate pathway inhibitor (Trimethoprim/sulfamethoxazole), MON: Monobactams (Aztreonam), PHA: Phosphonic acids (Fosfomycin), NIT: Nitrofurantoin.

**Table 6:** Patients’ characteristics by phenotypes classes.

Gender	Phenotype 4-1 (*n* = 39)	Phenotype 4-2 (*n* = 23)	Phenotype 4-3 (*n* = 23)	Phenotype 4-4 (*n* = 14)	*p*-Value
	% (*n*)	% (*n*)	% (*n*)	% (*n*)	
					0.007
Male	64.1 (25)	21.7 (5)	34.7 (8)	50.0 (7)	
Females	35.9 (14)	78.3 (18)	65.3 (15)	50.0 (7)	
Age (years)					0.349
<18	10.3 (4)	4.4 (1)	0.0 (0)	7.1 (1)	
18–60	43.6 (17)	56.5 (13)	73.9 (17)	57.1 (8)	
>60	46.1 (18)	39.1 (9)	26.1 (6)	35.8 (5)	
Healthcare service					0.137
Emergency	46.2 (18)	30.4 (7)	30.4 (7)	64.3 (9)	
Outpatient	53.8 (21)	69.6 (16)	69.6 (16)	35.7 (5)	
Trimester					<0.001
First	23.1 (9)	43.5 (10)	4.3 (1)	35.7 (5)	
Second	23.1 (9)	34.8 (8)	0.0 (0)	42.9 (6)	
Third	30.7 (12)	8.7 (2)	52.2 (12)	7.1 (1)	
Fourth	23.1 (9)	13.0 (3)	43.5 (10)	14.3 (2)	
